# PLX5622 did not alter anxiety-like behaviour and showed limited microglial depletion efficacy in a spinal cord injury model in female rats

**DOI:** 10.1038/s41598-026-48842-3

**Published:** 2026-04-22

**Authors:** Jaison Cucarian, Ram Rakheja, Kale R. Scatterty, Edward Armstrong, Sydney Storvold, Antoinette T. Nguyen, Keith Fenrich, Philippe Monnier, Karim Fouad

**Affiliations:** 1 Faculty of Medicine and Dentistry, Neuroscience and Mental Health Institute, University of Alberta, 8205 114 St NW, Edmonton, AB T6G 2E1 Canada; 2 Department of Physical Therapy, Faculty of Rehabilitation Medicine, University of Alberta, Edmonton, T6G 2G4 Canada; 3 Institute of Smart Augmentative and Restorative Technologies and Health Innovations (iSMART), University of Alberta, Edmonton, T6G 1G7 Canada; 4 Krembil Research Institute, University Health Network, Toronto, M5T 0S8 Canada; 5 Department of Ophthalmology and Vision Science, Faculty of Medicine, University of Toronto, Toronto, M5T 3A9 Canada

**Keywords:** Spinal cord injury (SCI), Microglia depletion, Colony-stimulating factor-1 (CSF1R), Plexxikon (PLX) 5622, Inflammation, Functional recovery, Neurology, Neuroscience

## Abstract

We previously reported that inflammation, when coupled with motor training following spinal cord injury (SCI) in female rats, promotes neuroplasticity as evidenced by enhanced performance in the trained task. However, this beneficial effect was accompanied by exacerbated anxiety-like behaviours, which are also a recognized consequence of SCI itself. Given that microglia are central mediators of the inflammatory response, their pharmacological ablation may help to clarify their specific role in enhanced motor recovery and their contribution to anxiety exacerbation. To this end, we first aimed to determine the efficacy and impact of the microglia-depleting compound PLX5622 in rats after SCI, as its effects in this traumatic injury model remain poorly characterized. The present study investigated how a four-week PLX5622 diet, started one week post-cervical SCI, influences anxiety-related behaviours, microglial depletion in the central nervous system (CNS), and SCI lesion pathology in adult female Lewis rats. Following the PLX5622 diet, no impact on anxiety-like behaviour was observed. The treatment achieved partial microglial depletion in all CNS regions examined, though to a lesser extent than reported in mice; no microglia morphological changes were detected in the brain or spinal cord. As expected due to delayed treatment, SCI lesion sizes were comparable across groups, but PLX5622-treated rats exhibited a less compact and thinner astrocytic scar at the lesion core. Importantly, our data revealed that PLX5622 also reduces resident macrophage populations in the liver, highlighting that its effects extend beyond the CNS. The limited impact of PLX5622 in our rat model of SCI, together with its lack of microglia specificity, raises concerns about its suitability for isolating microglial contributions to neuroplasticity, particularly regarding anxiety-like behaviour and post-injury motor recovery.

**Supplementary Information:**

The online version contains supplementary material available at 10.1038/s41598-026-48842-3.

## Introduction

Traumatic spinal cord injury (SCI) is a life-altering condition resulting from partial or complete spinal cord damage [1], leading to physical disabilities and decline in mental health [2]. The inflammatory response following SCI supports repair and recovery through the stimulation of neuroplastic changes [3]. Interestingly, the capacity for neuroplasticity after injury diminishes over time, similar to what is observed with inflammation. We previously demonstrated that inducing mild systemic inflammation with a lipopolysaccharide (LPS) injection, combined with a motor training task, enhances neuroplasticity and promotes recovery in rats with subacute [4] and chronic [5] SCI. However, these functional improvements were accompanied by persistent increases in anxiety-like behaviour [4], a phenomenon commonly observed after SCI and during systemic inflammation. Collectively, our previous work supports the dual nature of inflammation after central nervous system (CNS) injury. While it is essential for establishing a permissive environment for neuroplasticity and tissue repair, it can simultaneously drive maladaptive outcomes such as affective disturbances.

Microglia and macrophages, as the primary mediators of inflammation [6–8], have also been implicated in the exacerbation of anxiety [9]. Given their role, they represent a key therapeutic target for mitigating the detrimental consequences of inflammation post-SCI. Among the strategies to target these cells, their pharmacological inhibition is often the preferred approach. Compounds such as Plexxikon (PLX) 3397, PLX647, PLX73086, and PLX5622 inhibit the colony-stimulating factor 1 receptor (CSF1R). CSF1R is essential not only for the survival, proliferation, and proper function of microglia [10–12], but also for macrophages [13]. Among the CSF1R inhibitors, PLX5622 is the most widely used in preclinical research [14], due to its superior blood–brain barrier penetration, lower molecular weight, and higher specificity [14, 15].

Motivated by dual role of microglia and macrophages in modulating inflammation, we aimed to determine whether they may also be implicated in the improved recovery and increased anxiety in rats receiving LPS and motor training. Before addressing this question, however, it was necessary to establish the efficacy and specificity of PLX5622-mediated microglia depletion in rats, and its consequences on behaviour and spinal cord repair. By determining the scope of microglial depletion post-PLX5622 administration, we can ascertain whether this treatment will be suitable for testing the role of microglia in inflammation-mediated recovery and anxiety in future studies with our LPS and motor training treatment.

To evaluate PLX5622, female rats underwent partial cervical SCI and were treated with PLX5622 starting seven days post-injury, when secondary damage is largely resolved and would not be significantly affected by the treatment [16]. Anxiety-like behaviours were assessed throughout the PLX5622 treatment period. Following the *in vivo* phase, we evaluated PLX5622 depletion efficacy and specificity by quantifying residual microglia in the brain and spinal cord, as well as liver macrophages. Additionally, lesion size and glial scar thickness were assessed to determine whether microglial reduction impacts tissue damage.

We found that PLX5622 treatment in female rats with SCI induced only a partial microglial depletion within the CNS, with a more substantial reduction of liver macrophages. The incomplete CNS depletion may explain the absence of behavioural changes in the treated animals, with factors such as drug source, species-specific differences, and administration route being key determinants of PLX5622 efficacy.

## Methods

**Fig. 1:**
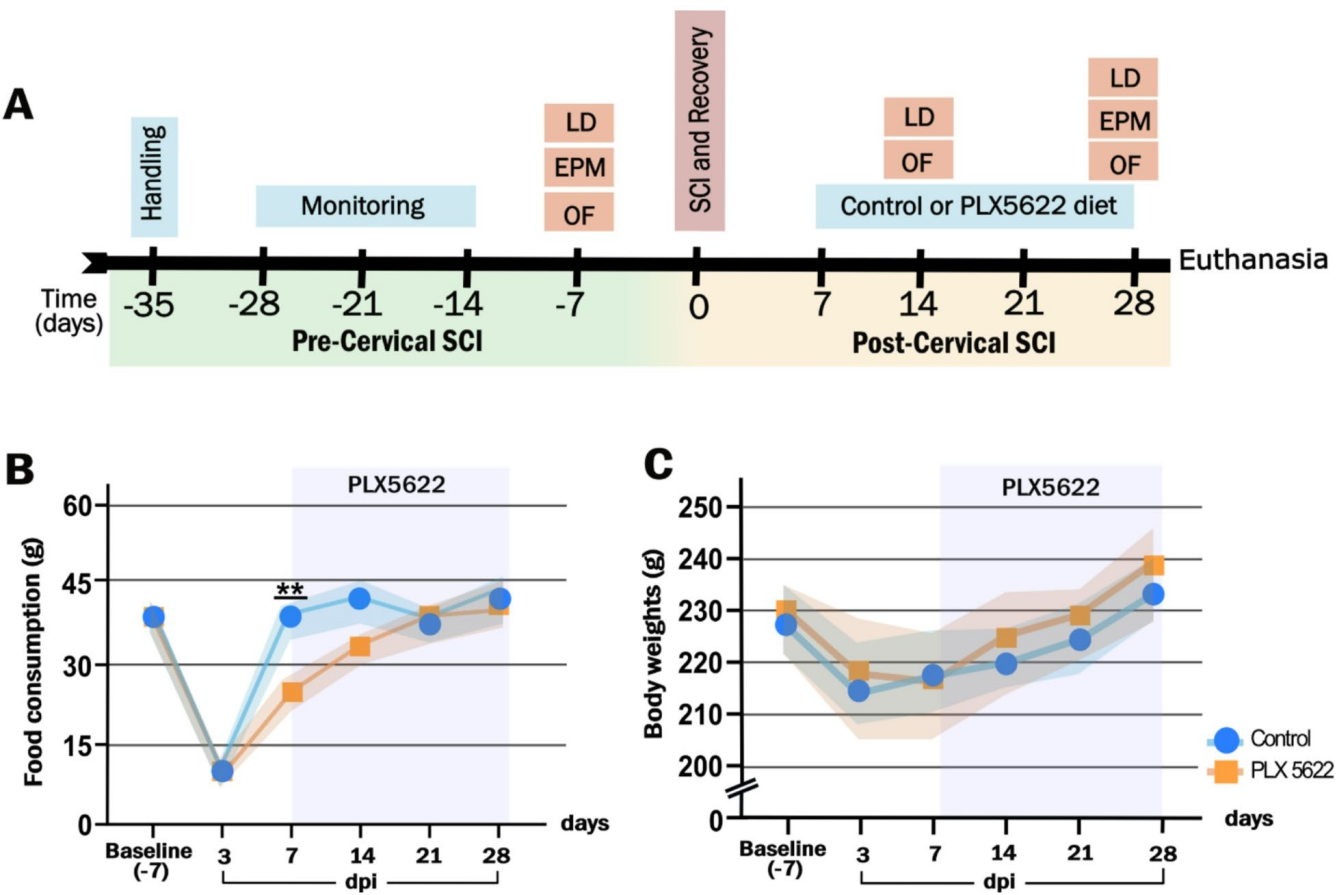
**Overview of experimental design with food intake and body weight monitoring.** (**A**) Experimental timeline showing behavioural assessments at weekly intervals before and after SCI, along with the 28-day PLX5622 treatment period post-SCI. (**B**) Food consumption is shown as the average daily intake per cage (3–4 rats), averaged weekly. (**C**) Body weight is represented as the average weight of rats per week. In panels B and C, the “baseline” time point corresponds to 7 days pre-SCI. Post-injury measurements are indicated as days post-injury (dpi), ranging from 3 to 28 dpi. The lilac-shaded area indicates the PLX5622 administration period. The blue circles and orange squares indicate the mean value in the group. The shaded areas in matching colors represent the SD of each group. **p = 0.001. Data were analyzed by two-way ANOVA followed by Holm-Šidák's multiple comparisons test. n = 7 animals per group. LD (Light–dark box test), EPM (Elevated plus-maze test), OF (Open field test), SCI (Spinal cord injury).

All animal procedures were approved by the Animal Care and Use Committee: Health Sciences at the University of Alberta and were conducted in accordance with the guidelines of the Canadian Council for Animal Care. This study is reported in compliance with the ARRIVE guidelines 2.0 (https://arriveguidelines.org). A detailed experimental timeline is provided in Fig. 1A.

### Subjects

To ensure comparability with our previous work [4, 5], and to avoid bladder expression challenges, we exclusively include female rats. Fourteen female Lewis rats (10–12 weeks old) weighing 180–220 g were obtained from Charles River Laboratories (Raleigh, NC). Animals were randomly divided and housed in groups of 3–4 per cage (50.8 cm long × 40.64 cm wide × 22.86 cm high). Environmental conditions were controlled and maintained at a temperature of 22–25 °C, humidity of 35–50%, and 12:12 h light: dark cycle. Food and water were provided *ad libitum*, with food weighed daily to determine consumption rates. Before experimental testing, animals underwent a 5-day handling period, 3 min per day between 8:00 AM and 11:00 AM, to minimize stress.

### Behavioural testing

Since microglia are known to modulate mood and affective behaviours without substantially impairing motor function, we prioritized the assessment of anxiety-like behaviour [10]. Three tests were conducted: the open field, elevated plus maze, and light–dark box, which also enabled for monitoring of locomotor activity. Behavioural assessments were conducted one week before SCI to establish baseline values in both groups, with follow‑up evaluations performed at weeks 2 and 4 of PLX5622 administration to monitor treatment effects over time. Before each test, animals were acclimated to the assessment room for 30 min. The equipment used for behavioural testing was cleaned between animals with an odorless detergent (Sparkleen) to remove olfactory cues that might affect the behaviour of subsequent animals. Behavioural tests were recorded with a camera (Panasonic Lumix, DMC-FZ200, resolution: 1280 × 720 pixels) placed directly above the testing arenas. Video analysis was conducted by a researcher who was experimentally blind to the treatment groups. For each test, animal behaviour was analyzed using a custom motion tracking software, Rat-Apps (version 1.0; available at: https://github.com/cdoolin/rat-apps) [17].

#### Open field test

As described previously by Schmidt et al. [17], the open field test was used to assess anxiety-like behaviour, such as exploratory and locomotor activity. Briefly, rats were individually placed in the center of a black acrylic arena (100 cm × 80 cm × 30 cm), the test was performed under dim illumination (100 lx). A five-minute trial was recorded per animal. Behaviour was assessed by measuring the distance traveled and exploration of the open field, specifically the time spent in the inner and outer zones.

#### Elevated plus-maze test

The elevated plus maze was utilized following the previously described protocol [17]. The maze was made of black acrylic and consisted of two open arms and two closed arms. Each arm was 50 cm long, 10 cm wide, and elevated to a height of 75 cm. The closed arms were enclosed by 40 cm-high walls on the sides and far end, while the open arms had no walls. The test was conducted under dim illumination (~ 100 lx). Each rat was placed in the center, facing one of the open arms, and allowed to explore the maze for 10 min. Spatio-temporal parameters, such as total distance traveled, entries into the open arms, and duration spent in each arm (open and closed), were analyzed.

#### Light–dark box test

Rats were placed into the dark compartment of a light–dark box apparatus (dark compartment: 0 lx; light compartment: 223 lx; each chamber: 30 cm long × 30 cm wide × 30 cm high) and allowed to explore for 10 min freely. The number of entries into the light box and the time spent exploring the light chamber were analyzed.

### Surgical procedure

All rats received a spinal cord dorsolateral quadrant (DLQ) transection injury, as previously described by Torres-Espín et al. [5]. Surgeries were performed under anesthesia with isoflurane (5% induction; 2.5–3% maintenance, supplied with a 50:50 air/oxygen mixture) and vital signs (heart rate and oxygen saturation) were monitored with a pulse oximeter. Eye lubricant was applied. After disinfecting the dorsal area of the cervical neck region with 10% chlorhexidine, a skin incision was made to expose the vertebrae. The interlaminar ligaments at C3-C5 in the cervical region were removed and a laminectomy was performed at the C4 vertebral segment. The dura was cut, and a unilateral DLQ transection was performed on the right side using a custom laser-cut blade with a 1.4 mm depth marker. Both muscle and skin were closed with absorbable sutures and surgical staples, respectively. The rats were closely monitored until awake. Each rat received a subcutaneous injection of 4 mL sterile 0.9% saline, and pain was controlled with buprenorphine (0.03 mg/kg; s.c.) for 1–2 days. After the SCI procedure, rats were allowed to recover for 7 days, during which their weight, water consumption, and food intake were monitored and recorded daily. Group assignment was based on pre-SCI behavioural test outcomes to ensure comparable baseline performance. Animals were then randomly assigned by an experimenter blinded to the study to either a control or a PLX5622 group.

### Diets

After 7 days post-SCI, a time when most of the secondary damage has occurred (Hellenbrand et al., 2024), control animals received a standard Rodent Diet 20 (n = 7) or PLX5622 (1200 PPM, MedChemExpress) incorporated into Rodent Diet 20 (PicoLab)(Research Diets, Inc., New Brunswick, NJ) (n = 7) for 28 days. PLX5622 was administered during the subacute phase to align with our LPS and motor training protocol [4, 5] and to avoid disrupting the acute injury phase. PLX5622 chow was stored at − 20 °C before being administered to the rats, following the manufacturer’s recommended handling instructions. Food was provided *ad libitum*. Body weight and food intake per cage were monitored daily.

### Perfusion and tissue processing

After 28 days on PLX, animals were euthanized with an overdose of sodium pentobarbital administered (100 mg/kg; i.p.). Rats were then transcardially perfused with a heparinized saline solution, followed by 4% paraformaldehyde (PFA) in 0.1 M phosphate-buffered saline (PBS). The fixative solution was supplemented with 5% sucrose to enhance tissue preservation and facilitate better fixative penetration. The brain, spinal cord, and the left lateral lobe of the liver were extracted and incubated in 4% PFA overnight at 4 °C. Afterward, the extracted organs were submerged in 30% sucrose for 1 week at 4 °C for cryoprotection. The brain was segmented into forebrain, midbrain, and hindbrain regions. A 5 cm section of the cervical spinal cord was collected and divided into lesion and non-lesion areas, each measuring 2.5 cm. The collected tissues were embedded in O.C.T. (Tissue-Tek) and frozen in 2-methyl-butane at − 60 °C using dry ice, and subsequently mounted onto filter paper. The tissue blocks were stored at − 80 °C. The brain, spinal cord, and liver were transversely sectioned at 25 μm thickness with a cryostat (Leica CM3050S, − 20 °C) and the tissue slides were stored at − 20 °C.

### Immunohistochemistry

The slides containing sections from the brain, spinal cord, and liver were warmed at 24 °C for 1 h. They were then rehydrated by rinsing them 3 times in 0.01 M PBS, followed by 3 washes in a solution of 0.01 M PBS containing 0.3% Triton X-100 (PBS-T), with each wash lasting 10 min. Slides were blocked for 1 h at room temperature (RT) with 10% normal goat serum (NGS, VectorLabs) or normal donkey serum (NDS, Sigma-Aldrich), depending on the species of the primary antibody host. To identify microglia, we used the pan-macrophage marker Ionized Calcium-Binding Adaptor Molecule 1 (Iba-1), which is also expressed by peripheral macrophages. Staining was performed using a rabbit anti-Iba-1 monoclonal antibody (1:1000, Cat. #019-19,741, Fujifilm Wako Chemicals, Osaka, Japan). Liver macrophages (Kupffer cells) were stained with a rabbit anti-F4/80 antibody (1:250, Abcam, #ab300421, Toronto, Ontario, Canada). To assess the extent of tissue damage and scar formation following SCI, microglia and astrocyte aggregation were evaluated using a rabbit anti-Iba-1 monoclonal antibody and a mouse anti-GFAP antibody (1:500, #G3893; Sigma-Aldrich, Missouri, USA), respectively. Tissue sections were incubated with the primary antibodies overnight at RT. The next day, the slides were washed 3 times with PBS-T for 10 min each. Tissue was incubated with secondary antibodies for 2 h at RT. The reaction products were visualized by Alexa Fluor (AF) 488 donkey anti-rabbit or anti-goat, AF 555 goat anti-rabbit or anti-mouse, and AF 568 donkey anti-rabbit (1:500). Finally, slides were coverslipped using Fluoromount-G with DAPI (# 00-4959-52, Invitrogen). Primary and secondary antibodies used in this study are detailed in (Table S1). Post-immunohistochemistry analyses were performed by a researcher blinded to animal group allocation.

### Imaging and microglia/macrophage quantification

The efficacy of PLX5622-mediated microglial depletion was assessed in five CNS regions. Three in the brain, the striatum, hypothalamus, and cingulate cortex, known for high baseline microglial density and involvement in affective behaviours, [18–21], as well as two cervical spinal cord segments (the intact C1 level and the injured C4 level). To verify that PLX5622 selectively targets microglia without affecting other immune cell populations, we quantified Kupffer cells as a control for potential off-target effects. Images for microglia/macrophage quantification were acquired using an epifluorescence microscope (Leica CTR 6000, Microsystems) at 20 × magnification (numerical aperture 0.50). A tile scan from 3 consecutive tissue sections from each area of interest was taken. Subsequently, a region of interest (ROI) was delineated using QuPath (Version 0.44; Software available at: https://qupath.readthedocs.io/en/0.4/) [22], based on the presence of 150 to 200 microglia/macrophage cells per section per animal in the control group. A semi-automatic quantification analysis in QuPath was applied to identify Iba-1- or F4/80- positive cells, ensuring that only cells with DAPI-stained nuclei were counted. A manual confirmation of the initial counting was performed using the imaging analysis software Fiji (Fiji Is Just ImageJ, Version 1.54 h; https://imagej.net/), following a previously described procedure [23].

### Microglial/macrophage morphometric analysis

For microglia/macrophage morphological analysis, a confocal microscope (Leica DMi8 and TCS SP8) with a 63 × oil immersion (numerical aperture 1.4) objective lens was used. Iba1-positive cells with DAPI-stained nuclei were captured from the analyzed brain and spinal cord structures of each animal. Within the regions assessed, multiple images from randomized locations were acquired to ensure a total of fifteen cells per animal for morphological characterization. The perimeter of each microglia soma was measured using the freehand selection tool in Fiji/ImageJ. Additionally, microglial arborization was analyzed by connecting the tips of each microglial process with lines drawn using Fiji/ImageJ’s polygon shape tool until all the processes were connected [23]. For morphological visualization, 3D reconstructions of microglia images were performed with Imaris software (Version 10.1; Oxford Instruments; available at: https://imaris.oxinst.com/versions/10-1). DAPI-positive microglia bodies were reconstructed using the “Surfaces” function on the selected ROIs.

### Lesion area analysis and glial scar thickness assessment

Analysis of lesion extension and glial scar thickness were performed at the lesion core, identified as the section with the most extensive damage, stained with GFAP. A confocal microscope (Leica DMi8 and TCS SP8) equipped with a 10 × objective lens (numerical aperture: 0.40) was used. Lesioned and spared areas were measured in µm^2^, and the extent of tissue damage was expressed as the percentage of spinal cord tissue affected within the analyzed section. Glial scar thickness, reflecting the astrocytic barrier around the lesion site [24], was quantified using GFAP immunofluorescence. Eight random measurements were taken along the glial scar and the mean was calculated for each animal.

### Statistical analysis

Statistical analyses were performed by an experimenter blinded to group allocation and independent of behavioural assessments and tissue processing, using GraphPad Prism (Version 10.1.2 for Windows; GraphPad Software, San Diego, CA, USA; https://www.graphpad.com/). Data normality was assessed using the Shapiro–Wilk test. Longitudinal data were analyzed by repeated-measures two-way ANOVA (after confirming normality) to evaluate group × time interactions, followed by Holm–Šidák-corrected post hoc tests for pairwise comparisons. For single-time-point variables, normally distributed data were compared using the unpaired t-test and are presented as mean ± standard deviation (SD). Non-normally distributed data were compared using the Mann–Whitney U test and are presented as median with interquartile range (IQR). Statistical significance was set at **α** = 0.05. Exact p-values are reported. All data were included in the analysis without exclusions.

## Results

### PLX5622 led to a decrease in food intake

In both groups (i.e., control and PLX treated), food intake decreased following SCI, with average weekly consumption per group declining from 39.2 g to 11.8 g, but returned to baseline levels within a week (Fig. 1B). Treated animals showed a significant reduction in food intake compared to controls during the first week of PLX5622 administration, consuming an average of 25.7 ± 2.3 g compared to 39.2 ± 7.2 g in the control group (Fig. 1B; (*F* (5,60) = 23.45; *t*_7 days_ (72) =  < 0.001; *p* = 0.001). No significant differences in food intake were observed between groups in the second (control: 41.7 ± 9.7 g; PLX5622: 33.8 ± 5.0 g; *p* = 0.19), third (control: 38.1 ± 6.1 g; PLX5622: 39.2 ± 7.2 g;* p* = 0.99), or fourth (control: 42.2 ± 9.9 g; PLX5622: 40.1 ± 8.0 g;* p* = 0.99) weeks after PLX5622 treatment onset.

Rats in both groups showed a slight decrease in body weight post-SCI. In the control group, the average body weight of a rat decreased from 226.7 ± 6.5 g at 7 days before SCI (day -7) to 214.0 ± 7.8 g at 3 days post-injury (Fig. 1C). Similarly, in the PLX5622 group, weight declined from 229.7 ± 6.26 g before SCI to 217.4 ± 11.3 g after SCI. The reductions in body weight observed in both groups were expected but did not reach statistical significance within either group (*p* =  > 0.99). Despite reduced food intake in the PLX5622 group during the first 7 days (Fig. 1B), body weight did not differ between groups, and both showed a gradual, slight increase at subsequent post-SCI time points.

### PLX5622 administration did not impact animal behaviour following SCI

**Fig. 2:**
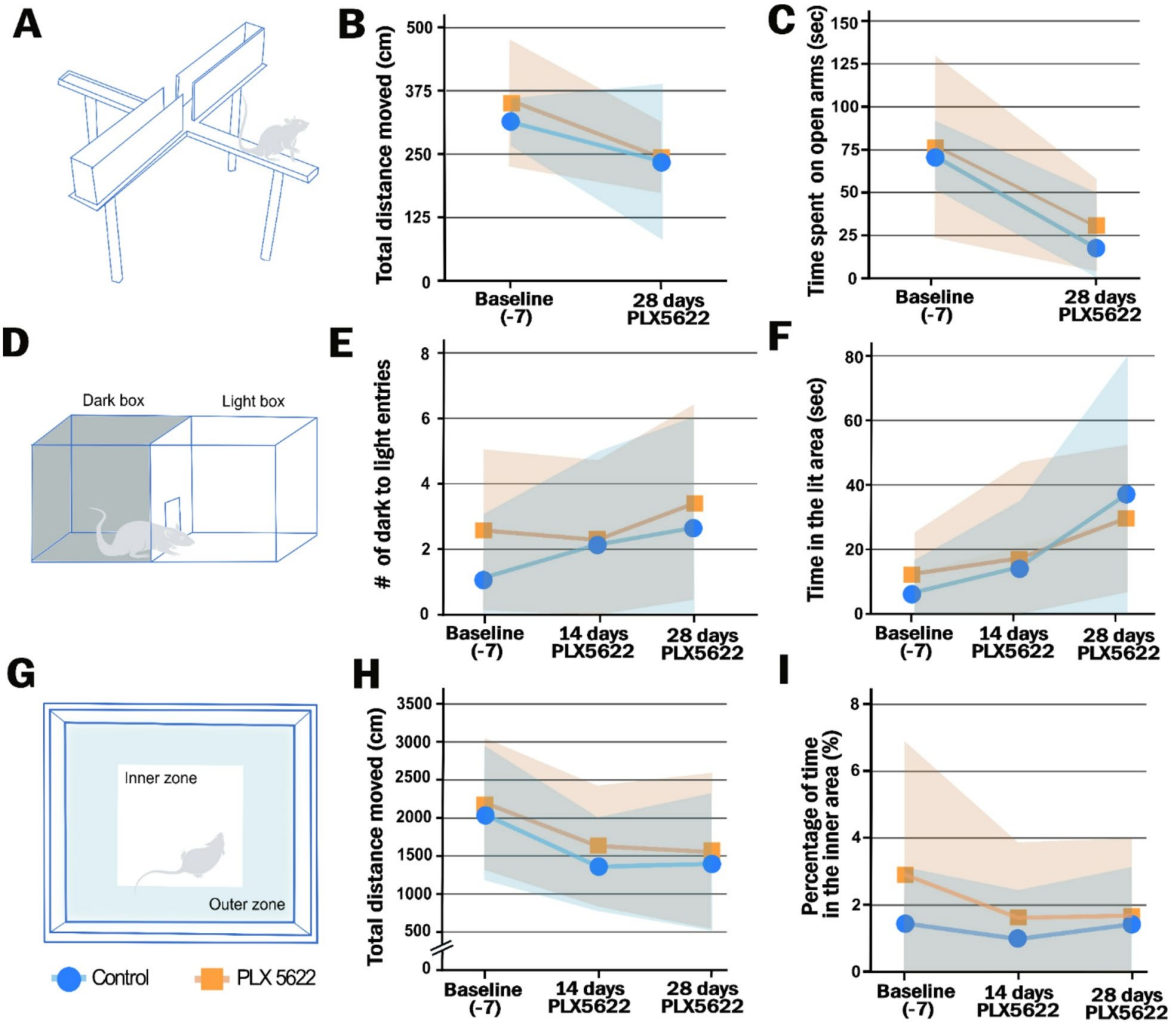
**PLX5622 did not impact anxiety-like behaviour or locomotor activity in rats following SCI.** (**A**) Elevated Plus Maze apparatus, where panels (**B**) and (**C**) show the total distance explored (cm) and the time spent in the open arms (sec), respectively. (**D**) Light–Dark Box arena, the number of dark-to-light entries is shown in panel (**E**) and panel (**F**) shows the average time each group spent in the lit box (sec). (**G**) Schematic Open-Field test, where (**H**) represents the total distance explored (cm) and (**I**) illustrates the corresponding percentage of the inner area explored (%). The blue circles and orange squares indicate the median value in the group at baseline (7 days before SCI), and at 14 and 28 days of PLX5622 administration. The shaded areas in matching colors represent the 25th and 75th percentiles interquartile range for each group. Behavioural data were analyzed by the Mann–Whitney U test. n = 7 animals per group.

A set of behavioural tests were applied to assess the effects of PLX5622 exposure on anxiety-like behaviour following SCI. Twenty-eight days of treatment with PLX5622 did not lead to any observable changes. In the elevated plus maze (Fig. 2A), rats in both groups exhibited a reduction in the total distance moved (Fig. 2B) and time spent exploring the open arms (Fig. 2C), and no significant changes were observed between the control and PLX5622-treated groups (*p* = 0.38 and *p* = 0.24, respectively).

In the light–dark box test (Fig. 2D), neither the number of dark-to-light entries (Fig. 2E) nor the exploration time in the light compartment (Fig. 2F) differed significantly between groups at 14 days (*p* = 0.98) or at 28 days (*p* = 0.51). The number of dark-to-light entries remained stable throughout the testing period. However, a slight, non-significant increase in time spent in the lit compartment was observed in both groups between days 14 and 28 of treatment.

Rats in both groups exhibited modest decreases in total distance travelled in the open field (Fig. 2G) following SCI; however, these reductions did not differ significantly between groups at either 14 days (*p* = 0.30) or 28 days (*p* = 0.52) (Fig. 2H). Similarly, the percentage of time spent exploring the inner area of the arena showed no significant between-group differences at 14 days (*p* > 0.99) or 28 days (*p* = 0.63) (Fig. 2I).

### Exposure to PLX5622 reduced partially the number of microglia in the brain and spinal cord

**Fig. 3:**
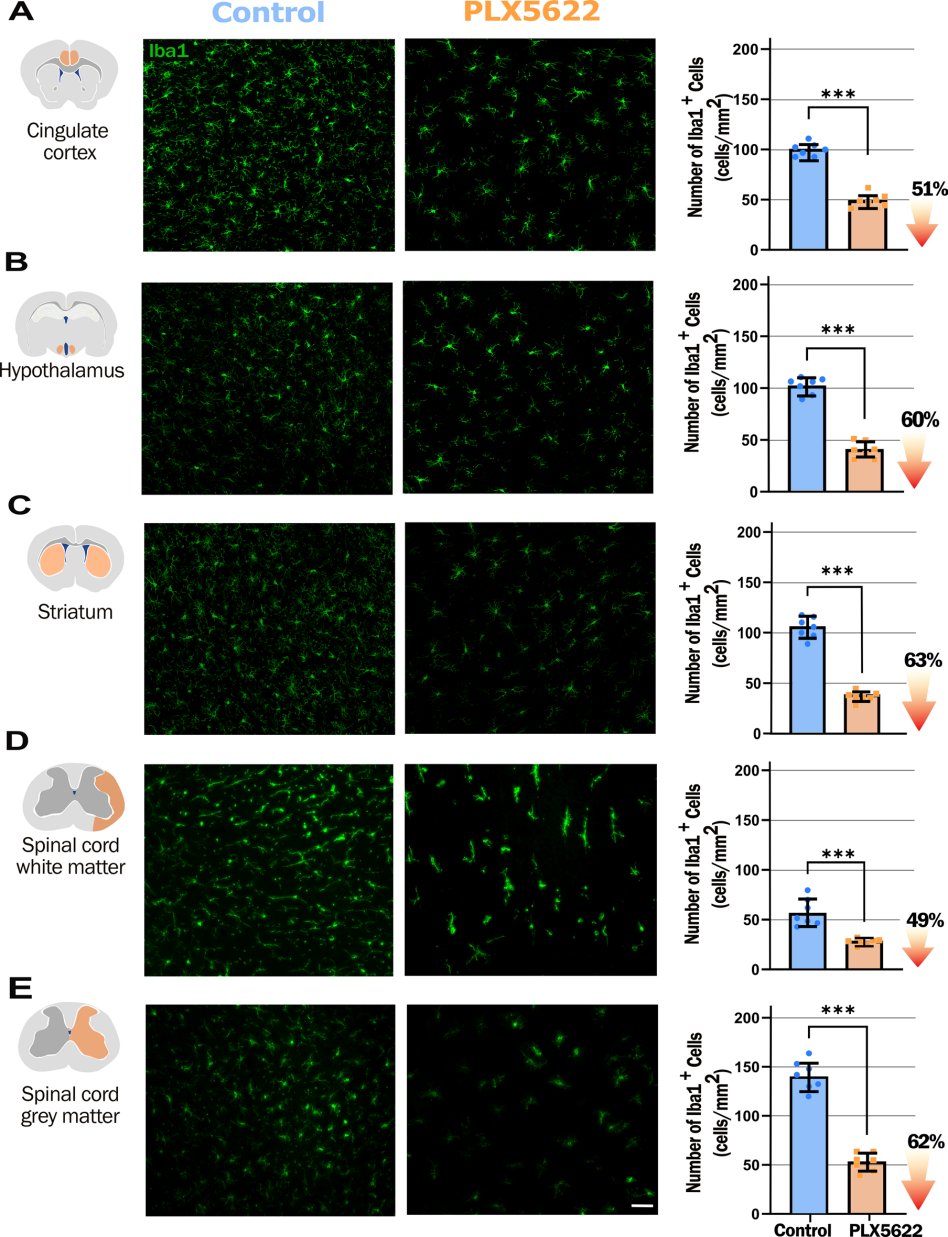
**PLX5622 reduces but does not deplete microglia in the nervous system of rats with SCI.** The orange-shaded regions in the schematic figures on the left correspond to the specific brain and spinal cord areas analyzed. Representative images from immunofluorescence staining of Iba-1-labeled microglia/macrophages and corresponding quantification in the (**A**) cingulate cortex, (**B**) hypothalamus, (**C**) striatum, (**D**) spinal cord white matter, and (**E**) grey matter from rats fed either a control or PLX5622 diet. Scale bar: 50 μm. Dots and squares in the bar graphs represent the average microglia/macrophage counts obtained from three quantified sections per region and animal. Bars represent the group median; error bars indicate the IQR. ***p ≤ 0.001. Comparisons between control and PLX5622 groups within each region were performed using the Mann–Whitney U test. n = 7 animals per group.

To test whether PLX5622 was effective at eliminating microglia/macrophages, Iba1⁺cells were quantified in various regions of the CNS. In the brain, the number of microglia/macrophages in the cingulate cortex showed the smallest reduction, with a median of 100.9 cells/mm^2^ (IQR: 85.1–136.2) in the control group and 49.4 cells/mm^2^ (IQR: 36.2–50.6) in the PLX5622 group (*U* = 0, *p* < 0.0006; Fig. 3A). The hypothalamus followed, with median values of 105.8 (IQR: 98.4–107.5) cells/mm^2^ in controls and 39.3 (IQR: 35.5–46.5) cells/mm^2^ in the PLX5622 group (*U* = 0, *p* < 0.0006; Fig. 3B). A slight increase in microglial reduction was observed in the striatum with a median value of 110.0 cells/mm^2^ (IQR: 95.1–117.5) in the control group compared to 38.0 cells/mm^2^ (IQR: 35.3–43.1) in the PLX5622 group (*U* = 0, *p* < 0.0006; Fig. 3C). A similar decline in microglia numbers was observed in the cervical region of the spinal cord, rostral to the lesion. When quantifying microglia/macrophages in the spinal cord, PLX5622 induced depletion in both white and grey matter. In the white matter, cell density decreased from 52.1 (IQR: 45.6–70.0) cells/mm^2^ in controls to 28.5 (IQR: 25.6–32.1) cells/mm^2^ in the PLX5622 group (*U* = 0, *p* < 0.0006; Fig. 3D). A more pronounced reduction was observed in the grey matter, where values declined from 142.0 (IQR: 129.8–150.6) cells/mm^2^ to 52.4 (IQR: 47.4–60.1) cells/mm^2^ (*U* = 0, *p* < 0.0006; Fig. 3E). In the analyzed regions, PLX5622 administration led to a 50–60% reduction in microglia/macrophage counts compared to control animals, with a coefficient of variation of 11.4%.

### PLX5622 did not exclusively affect immune cells within the nervous system

**Fig. 4:**
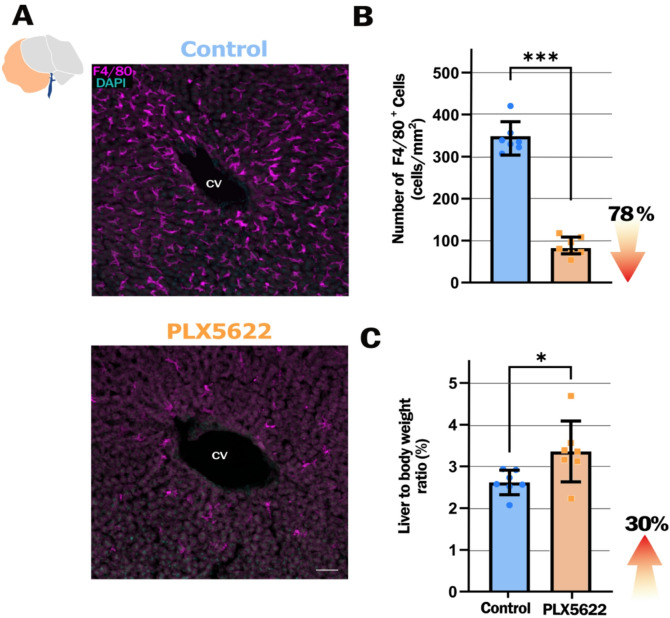
**Impact of PLX5622 on hepatic tissue.** (**A**) The analyzed liver lobe (left lateral lobe) is highlighted in orange in the schematic shown in the upper left corner. Representative confocal immunofluorescence micrographs show Kupffer cells (macrophages) stained with F4/80 antibody in control and PLX5622-treated groups. Scale bar: 50 μm (**B**) Kupffer cell density. Dots and squares represent F4/80-positive cell counts averaged from three quantified liver sections per animal. Bars represent the group median; error bars indicate the IQR. The percentage indicates the decrease in Kupffer cell number observed in the PLX5622 group (**C**) Liver-to-body weight ratio by group. Dots and squares represent individual ratios from animals in each group. Values are presented as median; error bars indicate the IQR. ***p < 0.0006; *p = 0.02. Group size: 7 animals per group. CV (Central vein).

To assess the ability of PLX5622 in targeting immune cells beyond the nervous system, we quantified macrophages in the liver (Fig. 4A), given their role in regulating the inflammatory response after SCI [25]. Quantification of Kupffer cells (hepatic macrophages) revealed a marked reduction in the PLX5622 group (median: 78.6 (IQR: 66.6–92.7) cells/mm^2^) compared to controls (median: 341.5 (IQR: 320.4–369.2) cells/mm^2^; *U* = 0, *p* < 0.0006; Fig. 4B), representing a 78% decrease in hepatic macrophage density. Additionally, by the end of the 28‑day treatment period, animals fed the PLX5622 diet exhibited a modest but significant increase in relative liver mass (liver‑to‑body weight ratio: 2.5% (IQR: 2.5–2.9) in controls vs. 3.3% (IQR: 3.1–3.5) in the PLX5622 group; *U* = 6, *p* = 0.01; Fig. 4C), corresponding to a 30% elevation in the ratio.

### PLX5622 did not alter microglia morphology

**Fig. 5:**
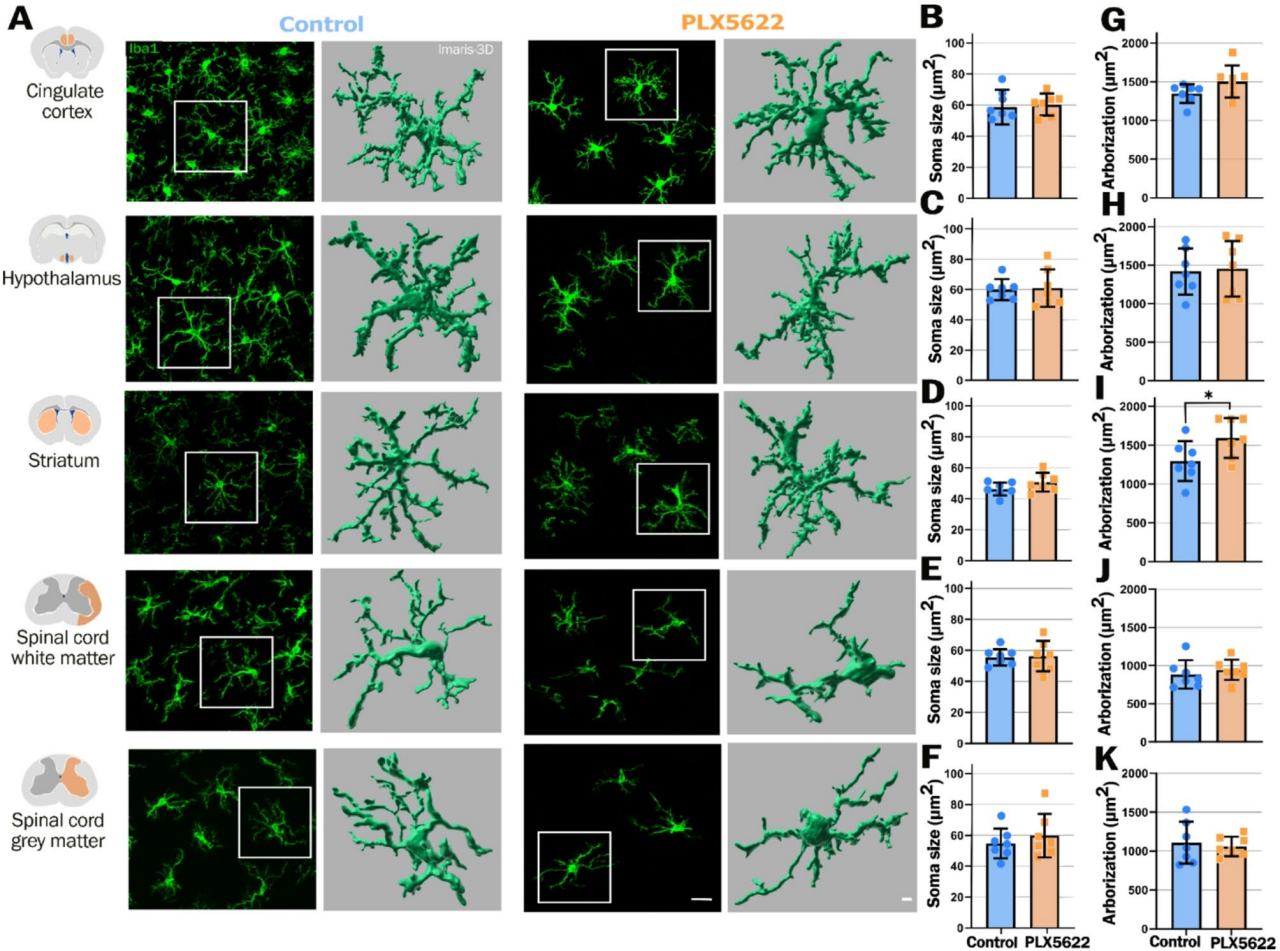
**Microglia morphological analysis.** (**A**) Schematic figures in the left column highlight in orange the brain and spinal cord regions analyzed. Representative immunofluorescence images to the right show Iba1-labeled microglia/macrophages and 3D reconstructions (for visualization purposes) in control and PLX5622 groups. Microglia/macrophage body size is illustrated in (**B**) Cingulate cortex, (**C**) Hypothalamus, (**D**) Striatum, (**E**) White matter, and (**F**) Grey matter of the spinal cord. Microglia arborization area is present in the (**G**) Cingulate cortex, (**H**) Hypothalamus, (**I**) Striatum, (**J**) White matter, and (**K**) Grey matter of the spinal cord. Scale bars: 20 μm for confocal images and 5 μm for 3D renderings. Dots and squares represent the average soma and arborization area per animal, calculated from 15 microglia/macrophage cells per animal. All values are expressed in square micrometers (µm^2^). Values are presented as mean ± SD. *p = 0.04. Comparison between groups were performed using the unpaired t-test. n = 7 animals per group.

To determine whether PLX5622 induces effects on microglia/macrophages beyond numerical reduction, the morphology of surviving cells was analyzed (Fig. 5A). Quantification of microglial soma size revealed no significant differences between control and PLX5622 groups across any region analyzed (cingulate cortex: *p* = 0.73; hypothalamus: *p* = 0.84; striatum: *p* = 0.13; white matter: *p* = 0.87; grey matter: *p* = 0.44; Fig. 5B–F). Similarly, arborization area did not differ significantly in the cingulate cortex (*p* = 0.10), hypothalamus (*p* = 0.83), white matter (*p* = 0.49), or grey matter (*p* = 0.67; Fig. 5G, H, J, K). However, PLX5622 treatment induced a significant increase in arborization area specifically in the striatum (control: 1296 ± 97.0 µm^2^ vs. PLX5622: 1594 ± 96.4 µm^2^; *p* = 0.04; Fig. 5I).

### Eliminating microglia did not influence lesion extension but resulted in a reduced glial scar after SCI

**Fig. 6:**
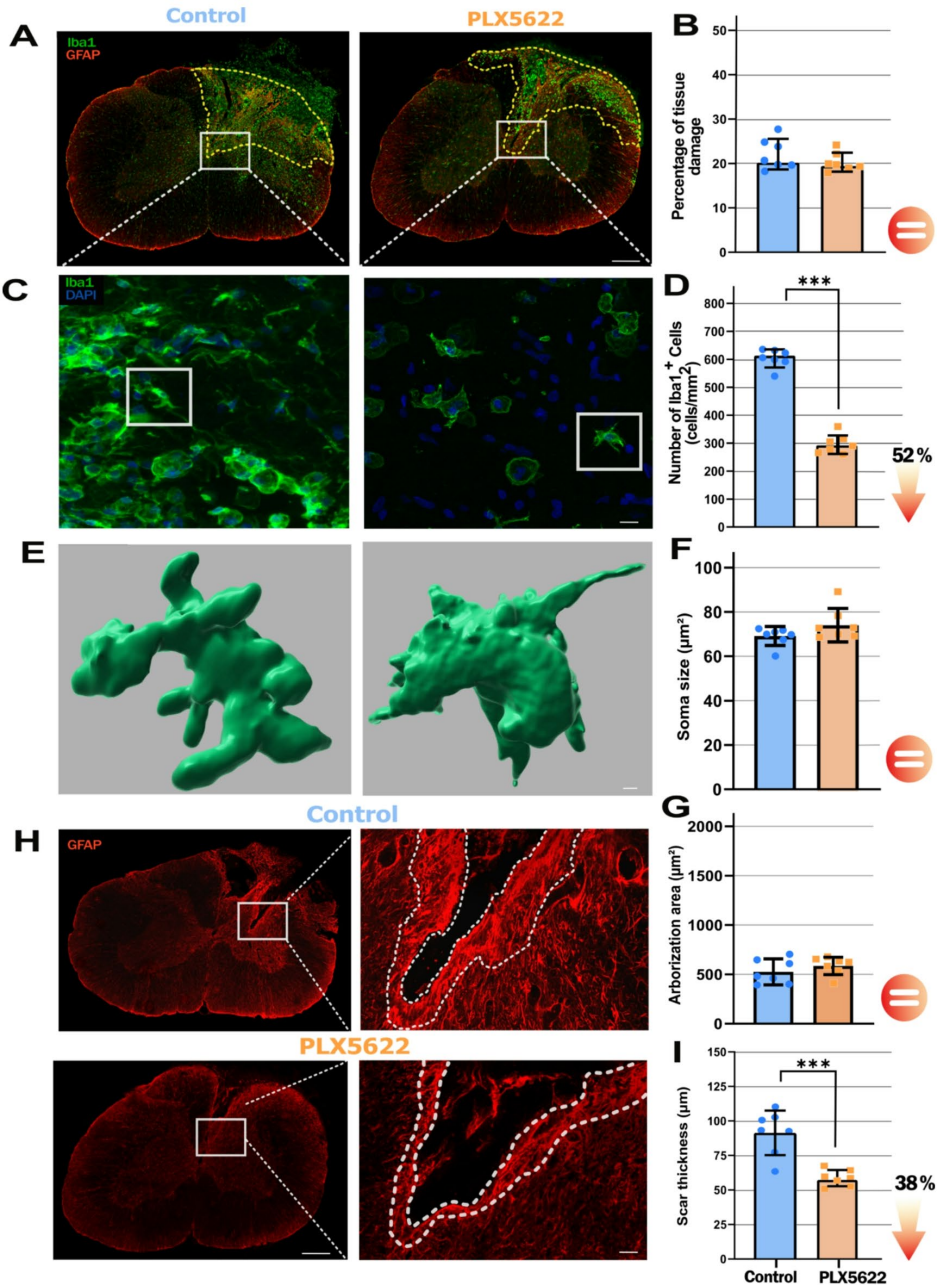
**Lesion size, microglia morphology, and scar thickness at the injury core.** (**A**) Representative immunofluorescence images of a spinal cord cross section showing Iba1 (green) and GFAP (red) labelling, with the lesion margin highlighted by a yellow dotted line. (**B**) The bar graph illustrates the percentage of lesion sizes across the groups. n = 7 animals per group. (**C**) Magnified view of Iba1 + microglia/macrophages at the lesion core. (**D**) Dots and squares in the bar graphs represent the average microglia/macrophage counts obtained from three quantified sections at the lesion core. n = 7 animals per group. (**E**) 3D reconstructions of microglia in the perilesional area (for visualization purposes). (**F**) Microglia body soma size and (**G**) arborization area measured around the lesion area, are expressed in square micrometers (µm^2^). (**H**) Representative GFAP staining illustrating astroglial activation and scar formation, delineated by a white dashed line. (**I**) Quantification of scar thickness in control and PLX5622-treated rats. Scale bars: 200 μm for spinal cord cross sections core, 5 μm for magnified images, and 2 μm for 3D renderings. Data are presented as mean ± SD for normally distributed variables and as median (IQR) for non-normally distributed variables. ***p < 0.0001. Statistical comparisons were performed using the Mann–Whitney U test for lesion size and microglia/macrophage counts, and unpaired t‑tests for microglial morphology parameters and scar thickness. n = 7 animals per group.

PLX5622 was administered 7 days post-SCI and requires approximately one week to deplete microglia/macrophages; by that time, secondary damage is expected to have abated. Nevertheless, lesion size was analyzed, as variations in lesion area could influence behaviour, inflammation, and microglial density. Additionally, thickness of the scar at the lesion core was assessed. For both purposes, astrogliosis and microglial reactivity were examined using GFAP and Iba1 staining. Quantification of lesion extent at the injury epicenter by GFAP and Iba1 immunoreactivity revealed comparable lesion sizes between groups (control: 20.6% (IQR: 19.5–24.8); PLX5622: 19.5% (IQR: 19.0–22.1); *U* = 15, *p* = 0.25; Fig. 6A–B). Given the dense microglia/macrophage clustering and tissue disruption within the lesion core, we focused our quantitative analysis on the peri-lesional white matter to ensure reliable cell number identification (Fig. 6C). Both groups exhibited a higher presence of Iba1-positive cells compared to other CNS regions analyzed (*see values on the Y axis in* Fig. 3). PLX5622 significantly reduced microglia/macrophage density at the lesion core (control: 614 (IQR: 579.5–643.9) cells/mm^2^ vs. PLX5622: 298.5 (IQR: 281.2–381.8) cells/mm^2^; *U* = 0, *p* < 0.0001; Fig. 6D), representing a 52% decrease relative to controls. The reduction in microglia/macrophages was consistent with that observed in other quantified CNS regions. Similar to other analyzed regions, average soma size at the lesion periphery was comparable between groups (control: 69.1 ± 1.6 µm^2^ vs. PLX5622: 74.0 ± 2.8 µm^2^; *t*(12) = 1.49; *p* = 0.19; Fig. 6F). However, microglial arborization around the lesion was reduced in both groups (control: 525.6 ± 49.5 µm^2^ vs. PLX5622: 586.2 ± 33.4 µm^2^; *t*(12) = 1.0, *p* = 0.32; Fig. 6G) compared to other CNS regions. Conversely, the size of the astrocytic scar, as measured by the thickness of the GFAP positive labelling around the lesion was thinner in the PLX5622-fed group (control: 91.3 ± 6.0 µm; PLX5622: 58.5 ± 2.2 µm; t(12) = 5.0, p = 0.0003; Fig. 6I).

Conversely, the size of the astrocytic scar, as measured by the thickness of the GFAP positive labelling around the lesion was thinner in the PLX5622-fed group (control: 91.3 ± 6.0 µm; PLX5622: 58.5 ± 2.2 µm; *t*(12) = 5.0,* p* = 0.0003; Fig. 6I).

## Discussion

As a preliminary study to evaluate the role of microglia in modulating motor recovery and anxiety within our LPS and SPRGR paradigm, this experiment assessed the effects of PLX5622-mediated microglia depletion on anxiety-like behaviour and spinal cord repair in female rats with SCI.

Inflammation after injury influences both recovery and the onset of mental health complications [8, 26], a dual role in which microglia are centrally involved. Microglia contribute to CNS function by modulating neuronal activity, supporting synaptic plasticity, and orchestrating complex processes underlying anxiety- and depression-like behaviours [27–30].

Previous research [31, 32], including our own [17], has established that SCI and inflammatory stimuli (e.g., LPS) [33] can increase anxiety-like behaviours in rodents, a finding supported in the present study that was not influenced by PLX5622. Although not statistically significant, our SCI-injured rats exhibited anxiety-like behavioural trends, characterized by reduced exploration of open zones in the elevated plus maze and open field tests. Conversely, a slight increase in lit-compartment exploration was observed during the light–dark box test. We attributed this discrepancy to animal habituation to the apparatus, a potential confound introduced by the short interval between repeated testing sessions. The absence of PLX5622 effects on behavioural outcomes may be attributed to two factors. First, although microglia are essential for CNS functioning under both healthy and pathological conditions [34], reducing their numbers may not substantially affect cognitive, motor, or affective domains in rodents (*for a comprehensive review see, reference* [10]). The absence of behavioural effects when microglia are depleted could be attributed to the fact that other CNS-resident glial cells, such as astrocytes, can partially compensate for the loss of microglial functions [35, 36]. Second, the extent of microglial reduction in our study may not have reached the necessary threshold to induce detectable changes in anxiety-like and locomotor behaviours.

In our study, administration of PLX5622 diet led to a reduction of Iba1 + cells by 49% to 63% across different regions of the CNS. However, the extent of microglia depletion observed in our study was less pronounced than that reported in previous research using adult rats and PLX5622. Most studies establishing the efficacy of PLX5622 for microglial depletion have been conducted in naïve animals. Indeed, sex-dependent variations in the depletion efficiency of PLX5622 have been reported, with females exhibiting more pronounced depletion compared to males [37]. In females, it has demonstrated a depletion of approximately 85–95% of microglial density in the brain after 7–12 days of treatment [37–39]. A similar level of microglia reduction in female rats has also been observed in mice, the most commonly used model for CSF1R inhibitors [15, 40–44].

The comparatively lower depletion in our study, despite the identical 1200 ppm dose, suggests the influence of methodological and biological factors. These include the source of PLX5622, the duration of its administration, the markers used for microglial identification, and even inherent differences between species. Earlier studies, including those ones here cited, used a specific formulation from Plexxikon Inc., which became unavailable after the company ceased operations in 2022. The PLX5622 used in our experiment was obtained from MedChemExpress, a formulation with variability in microglial ablation, with studies reporting effects ranging from widespread depletion [45, 46] to more limited efficacy [47]. Beyond the drug source, the duration of PLX5622 exposure is a critical determinant of microglia depletion efficacy. Although PLX5622 was administered for 28 days, treated rats consumed comparable food amounts to controls only after the second week (14 days), suggesting that longer exposure may be needed for greater microglial depletion. Nevertheless, studies in adult female rats have reported approximately 90% microglia depletion even after 7–10 days of PLX5622 administration [37–39], suggesting that 2 weeks may be sufficient to achieve substantial microglia ablation. Lastly, the lack of marker specificity in distinguishing microglia from peripheral macrophages could also contribute to the discrepancies observed. Sharon et al. [37–39], identified microglia in rats using co-labeling of Iba-1 with transmembrane protein 119 (TMEM119), a combination shown to better differentiate microglia from circulating macrophages [48, 49]. Our reliance on Iba1 alone, without TMEM119 or other co-labeling markers (e.g., P2RY12), represents a limitation in selectively distinguishing resident microglia from infiltrating peripheral macrophages. However, after SCI, both cell populations undergo a phenotypic shift [50, 51] that alters marker expressions [52] and reduces their specificity, even when combined markers are used. Furthermore, PLX5622 exerts systemic effects beyond the CNS, depleting macrophages in other organs, and potentially those found in circulation [53].

Microglia and macrophages have a high proliferative capacity, enabling them to repopulate nervous tissue within 3 to 7 days after PLX5622 withdrawal [12, 54, 55]. The marked proliferative capacity of microglia following CNS injury, coupled with the fact that not all microglia depend exclusively on CSF1R signaling for survival [56], may limit the efficacy of PLX5622. Together, these characteristics may provide an alternative explanation for our quantification results: surviving microglia could have rapidly proliferated to compensate, at least partially, for the initial depletion. However, this stands in contrast to studies in mouse models of CNS injury, which have demonstrated that PLX5622 remains effective in suppressing microglia even in the damaged CNS [15, 57, 58]. It has been observed that the pharmacological efficacy of some compounds can be species-dependent [59], and PLX5622 may not be an exception to this phenomenon. Nevertheless, to our knowledge, the specific mechanisms underlying the differing efficacy of PLX5622 in depleting microglia between species have not yet been explored.

When analyzing the impact of PLX5622 outside the nervous system, in our case in the liver, we observed a significant reduction of Kupffer cells (liver-resident macrophages). This finding aligns with previous studies reporting reduced resident macrophage numbers in other organs in mice following treatment with PLX5622 [41, 44, 53]. The depletion of Kupffer cells is not surprising, as other immune cells, such as monocytes, myeloid dendritic cells, and osteoclasts, also exhibit high expression of CSF1R [60]. Nevertheless, although depletion in other cellular populations has been documented, their implications in the regulation of affective, cognitive, and motor functions remain unclear. Interestingly, the 78% depletion of Kupffer cells, a rate that matches the high levels of microglial depletion (80–90%) reported in other studies, suggests that our PLX5622 was pharmacologically active but may have had limited blood–brain barrier penetration. While CNS penetration is known to vary across PLX compounds (e.g., PLX5622 vs. PLX3397; [14, 61, 62]), it remains unknown whether differences in the source of PLX5622 affect this property, although our findings suggest that they might.

Contrary to Sharon et al. [37], our results demonstrate that PLX5622 did not significantly alter microglial morphology in most non-lesioned CNS regions. Three factors could explain our findings: first, the use of distinct PLX5622 formulations from a different supplier; second, functional phenotypic shift occurring independently of visible structural changes; and third, differences in the methods used for microglial morphology assessment. Notably, the striatum was the only brain region where PLX5622 modified microglia arborization, increasing it. We proposed that the higher striatal microglial ramification may reflect region-specific variations in microglial susceptibility and density. The greater loss of microglia in the striatum compared to other CNS structures analyzed could lead the remaining microglia to extend their processes to increase their surveillance area [63]. However, whether longer microglial processes could affect striatal functioning remains unknown. Future studies employing 3D morphological analysis and single-cell transcriptomics will be necessary to fully characterize the impact of PLX5622 on microglial structure and function.

At the lesion core, no differences in the extent of tissue damage were observed, which was expected since the PLX5622 diet began 7 days post-injury, a time when neuroprotective effects were unlikely. Regarding microglial morphology around the lesion, we observed reduced arborization, consistent with a reactive state in response to SCI, as well as increased phagocytic activity and debris clearance [64–66]. The preserved microglia shape at the lesion site across groups, as in most CNS structures analyzed, suggests that the PLX5622 used here, may not substantially influence their functional properties. Lack of microglial physical changes influenced by PLX5622, however, needs to be taken carefully, as microglia morphology does not always mirror their functional states [52]. Interestingly, a thinner scar was observed in the PLX5622-treated group. Our observation aligns with previous studies reporting a key synergistic interaction between astrocytes and microglia in scar formation [67, 68], with microglia contributing by phagocytosing dead cells and debris, thus supporting glial scar establishment [69, 70]. While scar formation following SCI is often viewed as a structural barrier that limits axonal regeneration and functional recovery [71], an attenuated astrocytic scar may have additional detrimental consequences. A thinner scar could facilitate the spread of inflammatory and toxic mediators into spared tissue, thereby exacerbating and expanding tissue damage [67]. In addition, it may compromise the structural scaffold necessary for tissue repair and the regeneration of surviving axons [70].

A limitation of this study is the exclusive use of female rats, which was chosen to ensure compatibility with our previous work. We acknowledge that anxiety-like behaviours and neuroimmune responses differ substantially between sexes; therefore, future studies should evaluate the effects of PLX5622 in this regard and determine potential sex-specific impacts. Additionally, assessing anxiety after SCI in animal models is challenging, as motor impairments and the presence of pain can confound the expression of anxiety-related behaviours. Although our study did not include comprehensive evaluations of these spheres, we recommend that they be considered in future studies to avoid misinterpreting results when testing interventions with the potential to impact affective domains. Of particular relevance would also be characterizing the functional state of the remaining microglia and elucidating whether PLX5622 compromised their properties in ways that could influence recovery and anxiety-like behaviour post- SCI. Furthermore, the unknown proliferation rates of microglia and macrophages after SCI in the CNS, which could affect the perceived efficacy of PLX5622, meaning that continuous replenishment of these cells may make the treatment appear less effective. Like many studies in this field [72], we could not distinguish microglia from peripheral macrophages in our rats due to the lack of marker specificity. This distinction becomes even more challenging after SCI, which triggers complex and long-lasting immune responses, along with increased brain and spinal blood barrier permeability, allowing greater infiltration of peripheral immune cells [73].

Our study highlights important methodological factors for PLX5622 use in rats with CNS injury. We found that PLX5622 in female rats led to a partial depletion of microglia, contrasting with reports using Plexxikon’s PLX5622 in mice and rats. This discrepancy is unlikely due to the administration route, but may instead stem from differences in variations in drug batches, supplier, species, or animal strain.

## Conclusion

Our findings revealed that a 28-day administration of PLX5622 in female rats with cervical SCI only partially reduced microglial density without significantly impacting anxiety-like behaviours or tissue damage. While the lack of effect on anxiety, and spinal cord tissue can be seen as positive, as it suggests that PLX5622 does not interfere with motor performance in studies involving training. The partial microglia depletion makes it a less reliable tool to be used in our journey to investigate microglia’s role in the paradoxical effects of motor improvement and increased anxiety induced by our LPS and motor training paradigm. The efficacy of PLX5622 seems to be highly dependent on the compound source, species, sex, and administration protocols; this variability can lead to misleading outcomes and limit the reliability of PLX5622, underscoring the need for further research in the area. Importantly, the pronounced peripheral immune effects of PLX5622 emphasize that its actions cannot be interpreted solely from a microglia-centric perspective; off-target effects on other immune and non-immune cells must be considered. Furthermore, a deeper understanding of the species-specific mechanisms of PLX5622 is needed to resolve the discrepant findings across models and to elucidate microglia’s true therapeutic potential for recovery and mental health after SCI.

## Supplementary Information

Below is the link to the electronic supplementary material.

Supplementary Material 1

## Data Availability

All data obtained from this study is available from the Spinal Cord Injury Open Data Commons (10.34945/F5MS4M).

## Abbreviations

SCI: Spinal cord injury
CNS: Central nervous system
LPS: Lipopolysaccharide
CSF1: Colony-stimulating factor 1 receptor
PLX: Plexxicon
