# Supplementary material for: PLX5622 did not alter anxiety-like behaviour and showed limited microglial depletion efficacy in a spinal cord injury model in female rats
Source: Sci Rep. 2026 Apr 22;16:18749. doi: 10.1038/s41598-026-48842-3 (PMC13272775; doi:10.1038/s41598-026-48842-3)
Supplement: Supplementary file 1 — Supplementary Material 1 [file 41598_2026_48842_MOESM1_ESM.docx]

**Table S1. List of primary and secondary antibodies for immunofluorescence**

|  | **Host** | **Dilution** | **Source** |
| --- | --- | --- | --- |
| **Primary antibody** | | | |
| Iba-1 | Rabbit | 1:1000 | FUJIFILM Wako Chemicals, #019-19741 |
| Iba-1 | Goat | 1:300 | Novus Biologicals, #NB100-1028 |
| GFAP | Mouse | 1:500 | Sigma-Aldrich, #G3893 |
| F4/80 | Rabbit | 1:250 | Abcam, #ab300421 |
| **Secondary antibody** | | | |
| AF 488 anti-rabbit | Goat | 1:500 | Thermo Fisher, #A-11008 |
| AF 488 anti-goat | Donkey | 1:500 | Thermo Fisher, #A-11055 |
| AF 555 anti-rabbit | Goat | 1:500 | Thermo Fisher, #A-21428 |
| AF 555 anti-mouse | Goat | 1:500 | Thermo Fisher, #A-21237 |
| AF 568 anti-rabbit | Donkey | 1:500 | Thermo Fisher, #A-10042 |

### Abbreviations: Iba-1 (ionized calcium-binding adaptor molecule 1), GFAP (glial fibrillary acidic protein), and AF (Alexa Fluor).
